# A functional spiking neuronal network for tactile sensing pathway to process edge orientation

**DOI:** 10.1038/s41598-020-80132-4

**Published:** 2021-01-14

**Authors:** Adel Parvizi-Fard, Mahmood Amiri, Deepesh Kumar, Mark M. Iskarous, Nitish V. Thakor

**Affiliations:** 1 Medical Biology Research Center, Kermanshah University of Medical Sciences, Kermanshah, Iran; 2 Medical Technology Research Center, Institute of Health Technology, Kermanshah University of Medical Sciences, Kermanshah, Iran; 3 SINAPSE Laboratory, National University of Singapore, Singapore, Singapore; 4 Department of Biomedical Engineering, Johns Hopkins University, Baltimore, MD USA; 5 Department of Biomedical Engineering, National University of Singapore, Singapore, Singapore

**Keywords:** Neuroscience, Computational neuroscience, Sensory processing

## Abstract

To obtain deeper insights into the tactile processing pathway from a population-level point of view, we have modeled three stages of the tactile pathway from the periphery to the cortex in response to indentation and scanned edge stimuli at different orientations. Three stages in the tactile pathway are, (1) the first-order neurons which innervate the cutaneous mechanoreceptors, (2) the cuneate nucleus in the midbrain and (3) the cortical neurons of the somatosensory area. In the proposed network, the first layer mimics the spiking patterns generated by the primary afferents. These afferents have complex skin receptive fields. In the second layer, the role of lateral inhibition on projection neurons in the cuneate nucleus is investigated. The third layer acts as a biomimetic decoder consisting of pyramidal and cortical interneurons that correspond to heterogeneous receptive fields with excitatory and inhibitory sub-regions on the skin. In this way, the activity of pyramidal neurons is tuned to the specific edge orientations. By modifying afferent receptive field size, it is observed that the larger receptive fields convey more information about edge orientation in the first spikes of cortical neurons when edge orientation stimuli move across the patch of skin. In addition, the proposed spiking neural model can detect edge orientation at any location on the simulated mechanoreceptor grid with high accuracy. The results of this research advance our knowledge about tactile information processing and can be employed in prosthetic and bio-robotic applications.

## Introduction

Our ability to touch and feel is usually considered a simple task in daily life. However, there is a complex process behind our sense of touch, from the sensory receptors within the skin to the brain’s neuronal activity. When we interact with an object, the activation of different kinds of mechanoreceptors in the skin send various signals regarding object characteristics such as texture, shape, and size. Single-unit recordings of tactile afferents have been used to discover how tactile information is encoded^1,2^. One of the main findings is that stimulus information is distributed over a population of fibers in the form of spike trains. However, our understanding of population-level coding is still basic and only a few models have tried to address this important representation^3–5^. Indeed, tactile processing involves neural mechanisms that extract high-level features of a stimulus, such as an edge orientation, by integrating information from many low-level inputs^6,7^. The low-level inputs originate from the cutaneous mechanoreceptors which are located in the skin throughout the body and convert skin deformation into spiking responses. The density of mechanoreceptors varies across the body. For example, the density of cutaneous afferents that innervate the human fingertip is about 240 units/cm^2^, whereas in the palm it is only 58 units/cm^2^^8,9^. In general, the hands and lips for primates have the highest density of mechanoreceptors while the legs and back contain the lowest. One of the main features of these first-order neurons is that they have complex receptive fields. Indeed, the afferent’s distal axons which innervate the skin form many transduction sites^4,10^. This innervation pattern is a critical peripheral nerve mechanism that allows individual afferents to encode geometric information about tactile stimuli. To the best of our knowledge, the spatial complexity and heterogeneity of innervation patterns have not been integrated in models, such as in the excellent work by Saal et al.^3^.

The first stage of the tactile processing pathway consists of the cutaneous mechanoreceptors which are broadly categorized as two cell types with different response characteristics: (1) slowly adapting (SA) mechanoreceptors which produce sustained firing activity in response to a static indentation of the skin, (2) and rapidly adapting (RA) mechanoreceptors which mainly fire at the onset and offset of indentation. The SA-I and SA-II afferents innervate Merkel discs and Ruffini endings, respectively. The RA-I and RA-II afferents innervate Meissner and Pacinian corpuscles, respectively^11^. In this study, we consider SA-I and RA-I afferents which are essential for edge orientation detection^8^. The second stage in the tactile processing pathway is the cuneate nucleus (CN) within the medulla in the midbrain. The CN neurons incorporate lateral inhibition which filters neuronal firing. In this study, we investigate the role of lateral inhibition for edge detection at the population-level in the CN model^12^ by modulating the CN inhibitory currents. A mechanical skin stimulus activates several cuneate neurons, however, each cuneate neuron responds to a unique combination of inputs^13^. The spiking response of the second stage is transmitted (via the thalamus) to the cortical neurons in the somatosensory cortex of the brain (third stage) which are sensitive to edge-orientations and hence the stimuli are perceived^14,15^. Indentation at different skin locations evokes activity in different areas of the somatosensory cortex, with some overlap between neighboring skin regions^16^. Even though the collective response of the cortical neurons to the contact events looks very similar to the population response of afferents, tactile information is processed during transmission from the peripheral nerves to the cortical neurons. Indeed, cortical neurons have complex receptive fields with excitatory and inhibitory subfields and have properties that are largely absent in the tactile afferents^17^. These properties include feature selectivity to edge orientation^18^ and motion direction^19^ and nonlinear integration of inputs^6,20–24^. The three stages of the tactile processing pathway are summarized in Fig. S1. In this study, a three-layer biophysical model using a spiking neural network is proposed to functionally simulate the tactile processing pathway. This model takes into account the recent physiological evidence^4,12,17,18^. For the first layer, the SA-I and RA-I afferents are modeled as they branch into the skin and innervate overlapping receptive fields which provides spatial and temporal information about the tactile stimuli^4,9^. We propose a dynamic model for the SA-I afferents which consists of a Merkel-cell component (input current) and a neurite component (a derivative of input current). This novel model also shows better adaptability than the static one which only considers the Merkel-cell component. In our model, the first-order tactile neurons randomly innervate the mechanoreceptors available at the fingertip^4^. The second layer of the proposed spiking neural network models the *projection neurons* (PN) and *interneurons* (IN) to simulate the CN structure. The primary sensory afferents make excitatory synaptic connections with both PN and IN, while IN makes only inhibitory connections with the PN^12^. The third layer models a population of pyramidal neurons (PY) and cortical interneurons (c-IN). We focus on the somatosensory cortex area 3b which has previously been identified by linear spatial receptive fields with spatially separated excitatory and inhibitory regions on the skin^25^. In this case, the PY and c-IN receive excitatory synaptic connections from the PN. Additionally, the c-IN also have inhibitory synaptic connections to the PY. For simplicity, in our simulations, the cortical receptive fields consist of two subfield regions on the skin (excitatory and inhibitory) that are adjacent and tuned for a specific orientation. The receptive fields for each cortical neuron are distributed across the skin; consequently, edge orientation is recognized accurately and rapidly by the firing responses of the cortical neurons independent of the edge stimulus location. Finally, we explore the effect of afferent receptive field size on the encoding of the scanned and indented edge stimuli, based on the first spikes emitted by the cortical neurons (PYs). The entire spiking neural network model was built using the Brian2 simulator with Python version 3.65^26^.

## Results

The primary objective of this study is to simulate the tactile pathway from the glabrous skin of the fingertip to the cortical neurons in the somatosensory cortex area 3b in response to edge orientation stimuli. In this way, we discover how the receptive field size modulates information about edge orientation in the spiking pattern of cortical neurons. In the first layer, the SA-I and RA-I afferents are simulated which innervate their mechanoreceptors in a random sampling pattern. Edge stimuli activate a population of mechanoreceptors that send tactile information to the upper layers. The next layer, the CN, integrates and categorizes the coming information. We examine the role of lateral inhibition in the PN population and demonstrate how insufficient inhibitory currents lead to a failure in suppressing skin hypersensitivity. In the last layer, modeling the excitatory and inhibitory receptive fields of the somatosensory cortical neurons shows the classification of edge orientation independent of stimulus location. Two experiments are done; (i) Indented edge experiment: the stimulus vertically indents the simulated patch of skin. (ii) Scanned edge experiment: the stimulus indents and moves across the skin in a specific direction.

### First-order neurons

**Figure 1:**
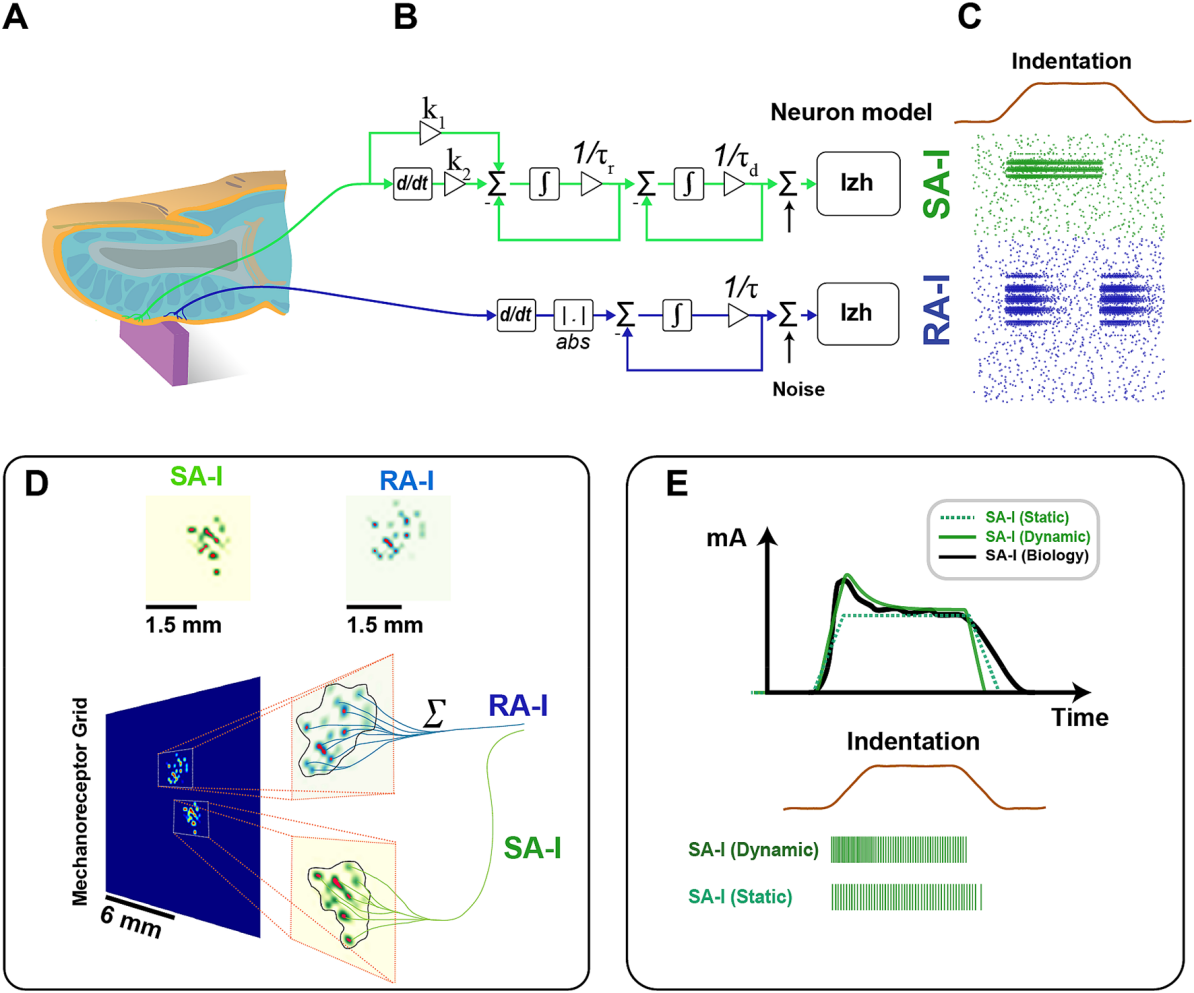
The first layer of the tactile processing model consists of a mechanoreceptor grid and two different populations of afferents (Green: SA-I and Blue: RA-I). (**A**) Touching an edge results in skin deformation, which in turn activates several mechanoreceptors distributed across the skin. (**B**) The signal processing pipeline for each afferent. (**C**) Simulated firing patterns of tactile fibers in response to an edge being indented into the skin at a 30° orientation. (**D**) Examples of receptive fields for the different mechanoreceptors. Each afferent randomly innervates a set of mechanoreceptors within their specific receptive fields. (**E**) The input current to the spiking neuron model (Izhikevich). The dynamic model of SA-I (green trace) produces spiking activity that is more similar to the reported physiological measurements^29^.

In the first layer, we simulated two afferent populations, namely, SA-I and RA-I. These afferents densely and randomly innervate the skin mechanoreceptors of the human fingertips and encode tactile information through their spike rates and spatiotemporal spiking patterns (Fig. 1A). A set of indentations are applied as the inputs to each afferent model (Fig. 1B) to produce spiking responses (Fig. 1C). The stimulus is the edge at different orientations (from 5°–80°, in 5° increments) indented on the mechanoreceptor grid of the fingertip. The different weights of mechanoreceptors make it possible for afferents to have spatially complex receptive fields (Fig. 1D). Recently, it has been proposed that random innervation of afferents might be a peripheral neural mechanism for extracting geometric features of the touched objects^4^.

#### The SA-I dynamic model

**Figure 2:**
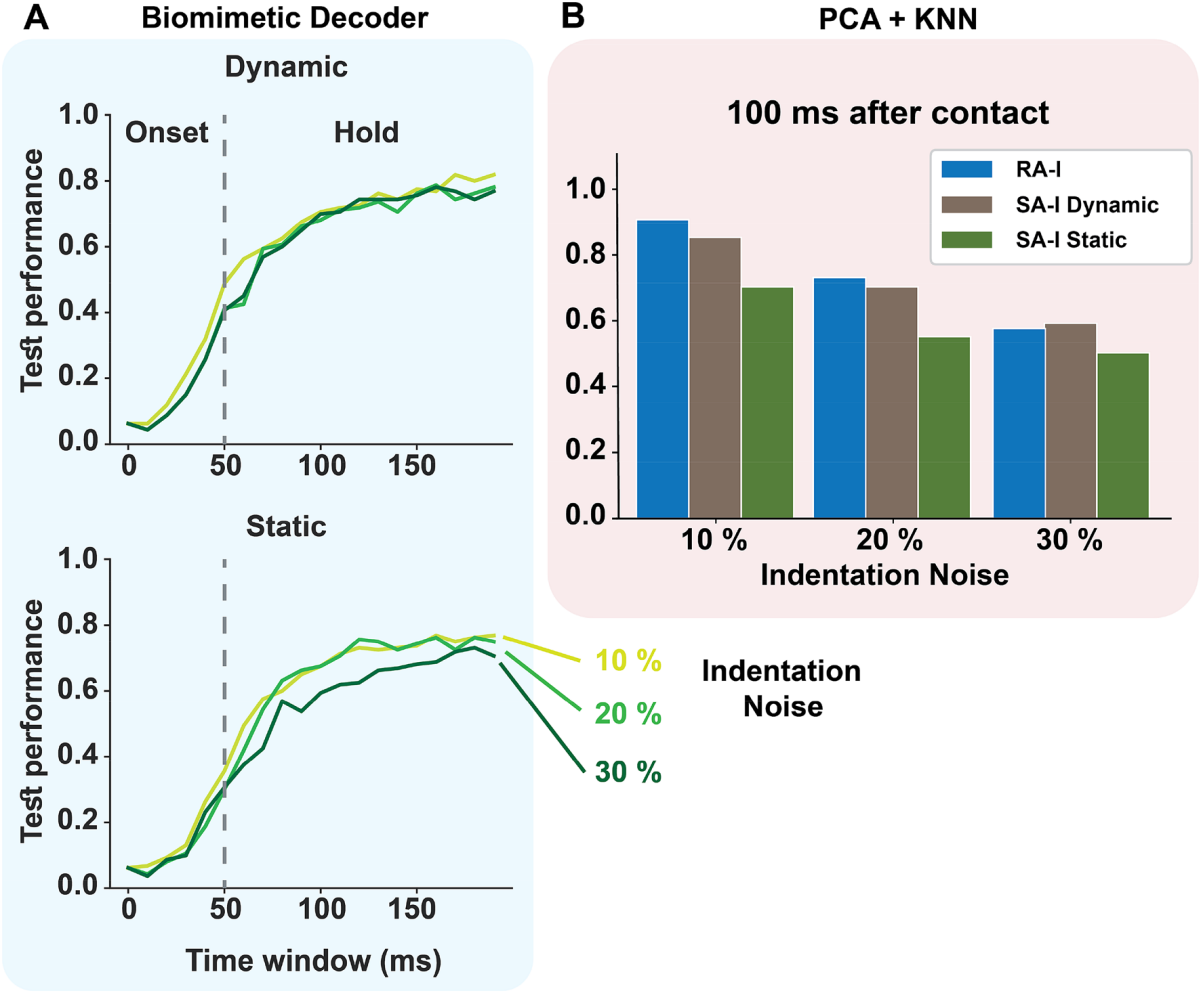
Network performance comparison for the static and dynamic models of SA-I afferent based on spike count analysis. (**A**) The biomimetic decoder is robust to noise for both static and dynamic models. (**B**) The KNN classification performance utilizing the first 3-principal components from afferent responses (RA-I, SA-I Dynamic, and SA-I Static models) to recognize orientation stimuli 100 ms after contact for three levels of indentation noise.

Myelinated SA-I afferents innervate the skin through unmyelinated fibers (neurites) to form synaptic-like connections with Merkel cells. Indeed, SA-I afferent’s end organ is considered as a Merkel cell-neurite complex^27^. When the skin is stimulated mechanically, Merkel cell-neurite complexes produce activation currents in unmyelinated neurites. Recent computational models have attempted to determine how structural mechanisms of Merkel cell-neurite complexes modulate the SA-I firing characteristics^28^, but it is still unknown how Merkel cells and neurites individually contribute to producing responses in the SA-I afferent. It is difficult to experimentally measure currents generated by the Merkel cells and neurites in response to a mechanical stimulation^27^. Nevertheless, recent biological data illustrate evidence for a two-component model. In this way, the Merkel cell-neurite complex includes (1) Merkel cells which are essential for sustained firing in SA-I afferents, and (2) neurites that produce rapidly adapting firing to mechanical stimulation^29–31^. Here, we propose a biologically plausible and functional model to mimic the Merkel cell and neurite response to mechanical indentation stimuli. Unlike previous studies which suggest that the SA-I model responds only to the hold phase of mechanical stimuli^32–34^, the new model is composed of a Merkel-cell component (input current) and a neurite component (a derivative of input current). The effect of the SA-I dynamic model on the performance of cortical neurons using spike count analysis is illustrated in Fig. 2. Considering indentation noise in different trials, it is found that the classification performance has a stable characteristic when a biomimetic decoder^35^ (see Methods) is used (Fig. 2A) and consequently it has more robustness. Afferent responses are assessed using Principal Component Analysis (PCA). In this way, considering the first 3-principal components of the feature space (spike count), simulations show that the K-nearest neighbor classifier (KNN) has higher performance for the SA-I dynamic model in comparison with the SA-I static model (Fig. 2B). Furthermore, the RA-I afferent responses against indentation noise are also reported in Fig. 2B. Noteworthy, the number of RA-I afferents is almost double of the SA-I ones (see Methods) and its performance is slightly superior. This figure also illustrates that the initial spiking responses of the SA-I model make rapid and accurate orientation detection.

#### Decoding the indented and scanned edge orientation

**Figure 3:**
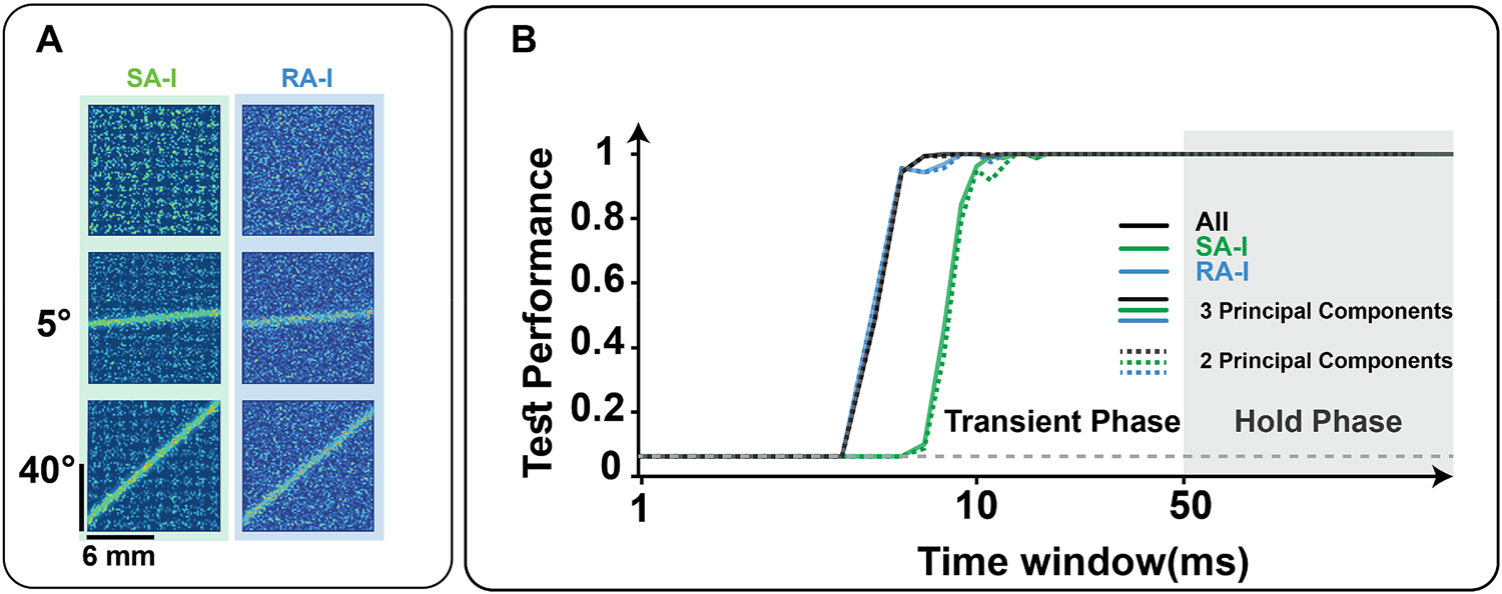
Decoding the orientation of indented edge based on spike count analysis. 16 orientations were tested (5°–80° with 5° steps). (**A**) The indented edges at 5° and 40° activate the mechanoreceptor population. The Left and Right columns indicate the mechanoreceptor grids innervated by SA-I and RA-I afferents, respectively. (**B**) Classification performance for SA-I (green) and RA-I (blue) afferents and the whole population (black) when the edge stimulus indents skin for different durations right after the contact. The PCA algorithm is used for dimensionality reduction and solid curves show the test performance when three principal components are considered. The chance level is shown by the horizontal gray dashed line.

**Figure 4:**
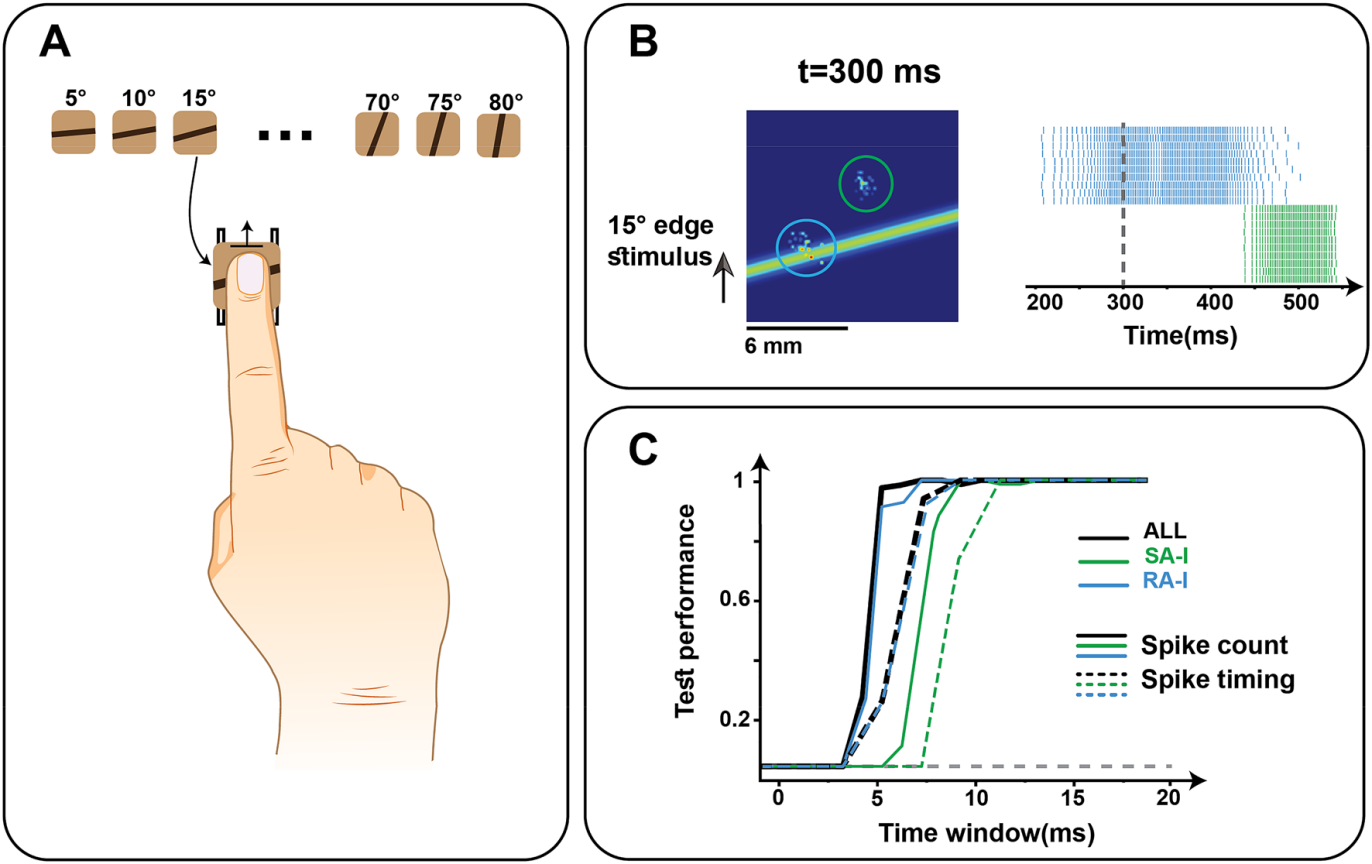
Decoding the orientation of a scanned edge. (**A**, Upper) The 16 edge orientation stimuli (5°–80° in 5° steps). (**A**, Lower) Simulated scanning edge experiment: The fingertip makes contact with the edge oriented at 15° and then the edge is moved upwards. (**B**, Left) Sampled mechanoreceptors which are innervated by two afferents (green circle shows the SA-I receptive field and blue circle indicated the RA-I one) and scanned edge moves across the receptive fields. In this way, the activated mechanoreceptors generate spiking responses in their corresponding fibers. (**B**, Right) Responses of 2 selected SA-I and RA-I fibers to the scanned edge stimulus for 10 repetitions. The SA-I and RA-I firing responses are depicted in green and blue, respectively. The vertical dashed line represents the time of edge location shown in (**B**, Left). (**C**) Classification performance for each afferent type (green and blue) and both (black) for different time windows using spike count (solid curves) and temporal metric (dash curves). The horizontal gray dashed line denotes the chance level.

In the indentation experiment, we investigated how edge orientation could be decoded and extracted from the SA-I and RA-I population responses. We consider 2 or 3 principal components obtained by applying PCA to the spike counts calculated from firing patterns. In our simulations, the Pacinian afferents have not been considered because they are known to contribute more to texture and vibrotactile encoding^3,8^. In Fig. 3A, as the orientation changes, the edge stimulus lands on the receptive field of different afferents, therefore, information is encoded in the spatiotemporal pattern of activation. Results show that contact stimuli are encoded in the first emitted spikes, and 2-principal components are as informative as 3-principal components which are extracted from the feature space (Fig. 3B). At the population level, the performance of RA-I is higher than SA-I given the fact that the number of RA-I afferents is greater than SA-I which improves the spatial encoding. Based on the experimental evidence, each type of afferent extracts particular geometric features, and therefore, the combination of both afferents improves the recognition performance. We also simulated the responses of two afferent populations to the scanned edge stimulus on the simulated patch of skin for 16 different orientations (Fig. 4A). The goal is to classify the edge orientation based on the responses at the population-level considering both spike count (number of spikes) and temporal spiking pattern (spike timing). Indeed, afferents randomly innervate the mechanoreceptors and create highly sensitive areas that are non-uniformly distributed within the receptive field^4^. This arrangement acts as a peripheral neural mechanism that allows individual neurons to extract distinct features of the touched objects. When skin is deformed, each afferent produces unique responses and facilitates edge stimulus discrimination. To calculate the temporal distance between spike trains, Victor-Purpura distance (VPd) has been used^36^.

Figure 4B shows the neural response when an edge stimulus is moving across the receptive fields of two sampled tactile afferents. In Fig. 4C, as can be seen, the RA-I responses rapidly show the orientation stimuli with high accuracy compared to the SA-I responses. Figure 4C shows the comparative performance analysis for decoding the orientation of the scanned edge to variable time windows. Both spike count (spatial activation pattern) and spike timing-based features are used for orientation detection. Although the classification accuracy for both methods, spike count and spike timing, eventually saturates, the performance curves of the spike timing method (dashed lines) have a delay with respect to the performance curve of the spike count method (solid lines). This interestingly shows the spatial activation pattern (which is specified by counting the number of spikes) of the early spikes conveys more information compared to the temporal patterns of the afferent responses.

**Figure 5:**
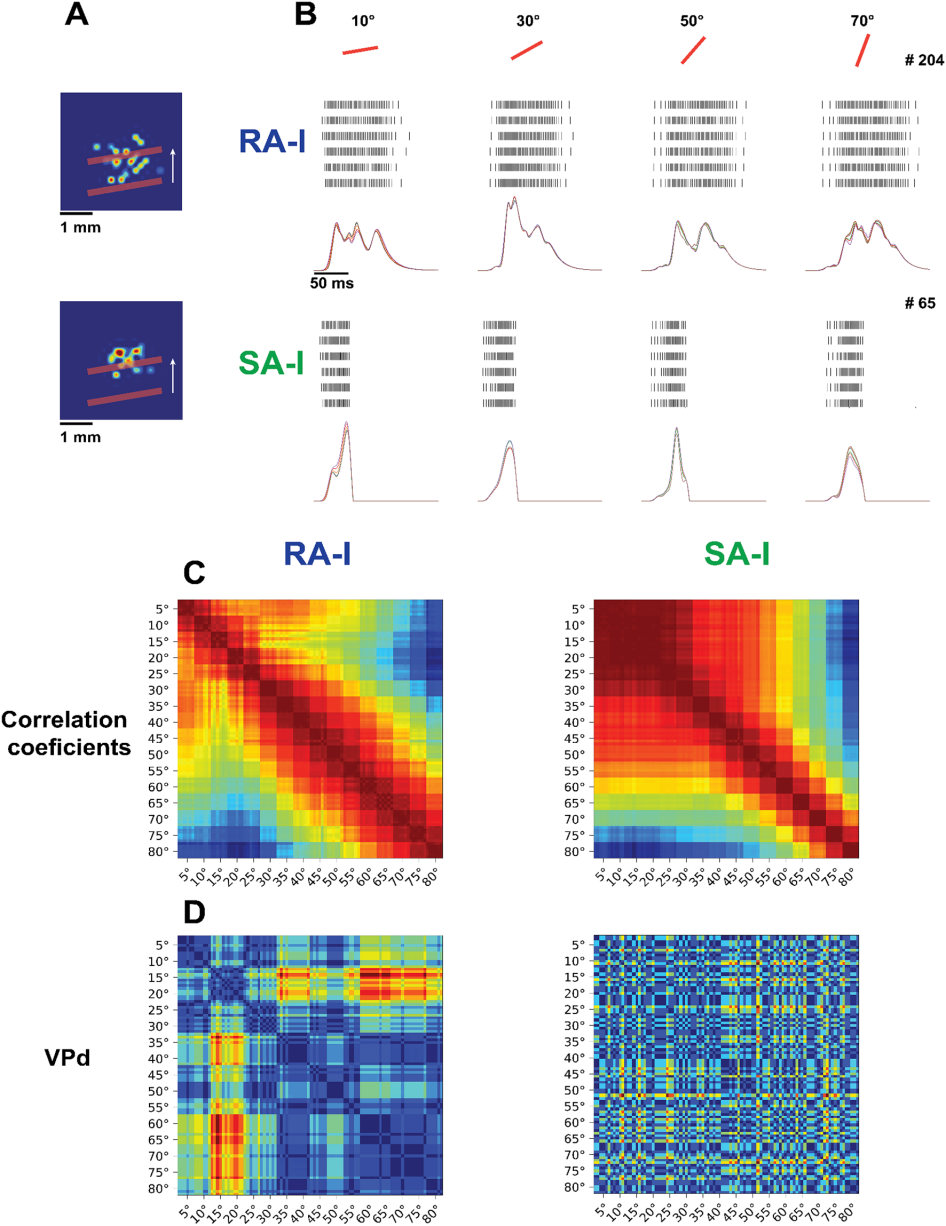
The firing responses of sampled SA-I and RA-I afferents for different edge orientations. (**A**) A line stimulus moves across the receptive fields of two primary afferents (Upper, RA-I (#204); Lower, SA-I (#65). (**B**) Raster plots and the generated currents for two neurons shown in A for four different orientations (10°, 30°, 50°, 70°) and 6 passes across the receptive field. (**C**) Correlation coefficients of the currents in mechanoreceptors for all 16 orientations scanned 6 times with 10% indentation noise per trial. (**D**) Victor-Purpura distance (q = 1) was calculated for each pair of spike trains. The extracted features from (**C**) and (**D**) are later used to classify the stimulus orientation.

To assess the behavior of the afferents at a single-unit level, one afferent (SA-I and RA-I) is considered which has its receptive field (Fig. 5A). During the experiment, the receptive fields are scanned by 16 edge orientations and each stimulus is repeated 6 times (96 trials in total). Since RA-I afferents have larger receptive fields in comparison to SA-I, the obtained spiking responses are more diverse (Fig. 5B). Figure 5C shows the cross-correlations between the input current of afferents. As can be seen in Fig. 5C, for the SA-I afferents, in lower angles of orientation, correlation coefficients are more similar. Thus, angle discrimination using correlation coefficients in RA-I is easier than SA-I. Figure 5D illustrates the spike timing analysis by computing the VPd metric which illustrates that the RA-I spike responses are more informative than SA-I. Employing the extracted features from Fig. 5C,D, the classification accuracy to classify the edge orientation is reported in Table 1.

**Table 1:** Classification performance of the sampled afferents for the scanned edge experiment across their receptive fields: taking into account the spike distance (VPd) and correlation coefficients as the input features to the KNN classifier.

Primary afferent	Spike distance (VPd) (%)	Correlation coefficients (%)
$RA-I$	$66$	$97.5$
$SA-I$	$15$	$88$

### Second-order neurons (cuneate nucleus)

**Figure 6:**
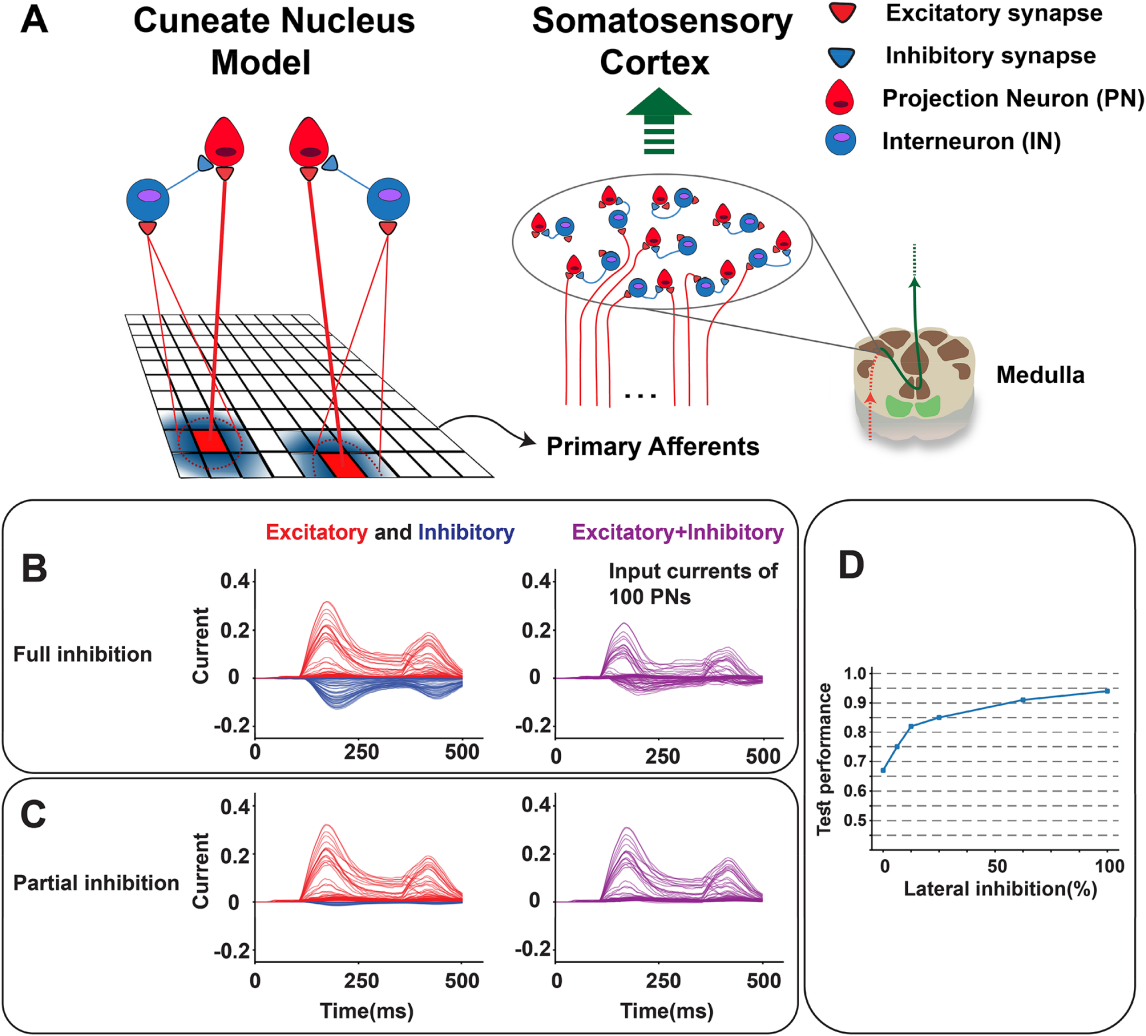
Modeling the CN neuronal circuitry and synaptic currents (excitatory and inhibitory) for edge indentation stimulus. (**A**) The primary afferents make excitatory synaptic connections with both PN and IN sub-populations. Their synaptic types are indicated by red (excitatory) and blue (inhibitory) triangles. Input currents of 100 PNs when full (**B**) and partial (**C**) inhibition comes from INs to PNs while edge stimulus indents the mechanoreceptor grid. Two peaks in the current traces are generated by the onset and end of the indentation profile. (**D**) Performance of the spiking network when the amount of lateral inhibition is increased within the CN model.

**Figure 7:**
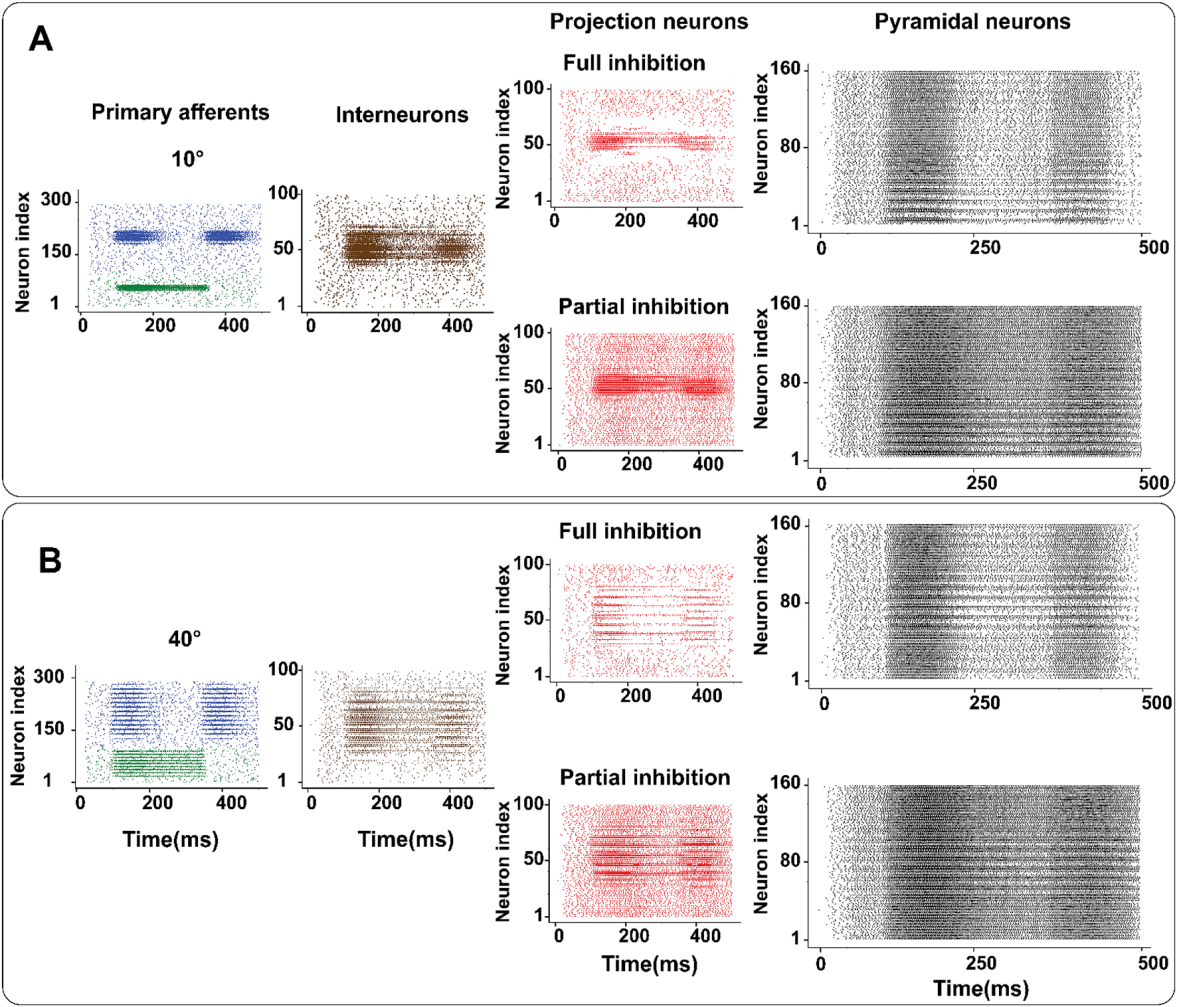
Spiking responses of different stages of tactile processing pathway when lateral inhibition in CN is modulated. (**A**, **B**) Raster plot of primary afferents (SA-I and RA-I), IN and PN subpopulation of CN and PYs for 10° (top) and 40° (bottom) indented edge stimuli. The filtering effect applied by the CN model for the full inhibition case is clear which facilities the recognition of the edge orientation. The amount of partial inhibition is 25%.

The second layer of the proposed functional neuronal model considers the *projection neurons* (PN) and *interneurons* (IN) to simulate the cuneate nucleus (CN) structure in tactile sensing. The CN neurons mainly contribute to tactile information processing through lateral inhibition which leads to the firing rate filtering^12^. The INs make inhibitory synaptic connections with the PNs (Fig. 6A). We have hypothesized that the lateral inhibition in the CN model prevents the accumulation of noise and preserves spatial information which in turn facilitates the edge detection process. In this way, the impact of CN lateral inhibition on the performance of orientation detection is explored when the edge indents the simulated mechanoreceptor grid. Indeed, we tune the network for the best performance of edge orientation detection. With maximum lateral inhibition, input currents of some PNs decrease significantly. On the other hand, reducing the amount of inhibitory current from INs to the PNs (Fig. 6B,C), leads to an increase in the firing rate of the cortical neurons. A biomimetic decoder is considered by employing the winner-take-all algorithm (a cortical neuron with a maximum firing rate is the winner neuron) (see Methods). Figure 6D illustrates that diminishing the amount of lateral inhibition leads to a decrease in the recognition accuracy of the indented orientations. Indeed, lateral inhibition preserves spatial information and thus facilitates the edge detection process. Firing responses of all tactile processing stages are plotted in Fig. 7A,B, when two edge orientation stimuli indent the mechanoreceptor grid at two levels of inhibition in the CN.

### Cortical neurons in the somatosensory area

**Figure 8:**
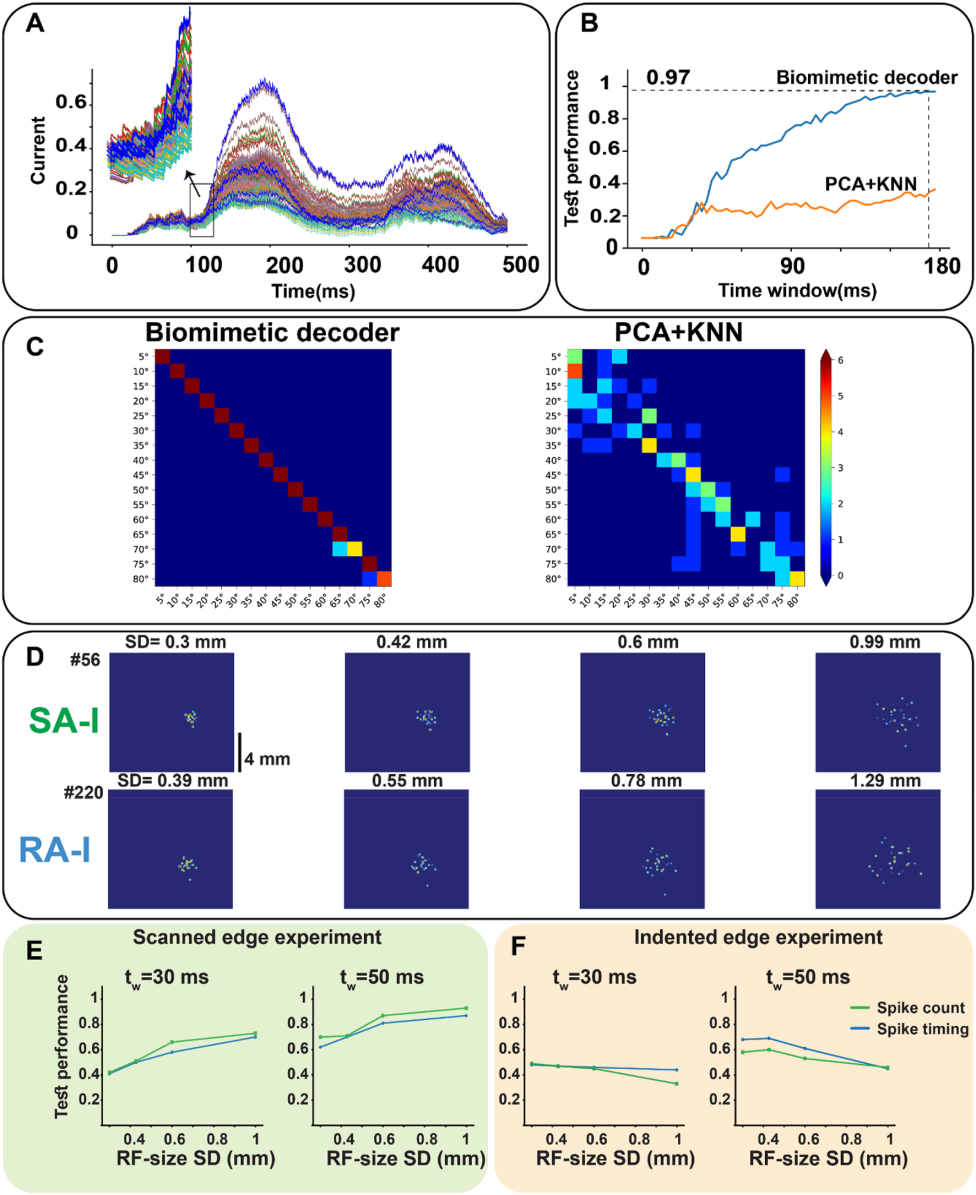
The performance of the tactile spiking neural network when stimulus location is changed on the skin. (**A**) Input currents of cortical neurons: blue traces show those neurons that are tuned to the presented input stimulus, each of them responds to a stimulus which is applied at a different skin region. The magnified part indicates the transient phase of indentation. Using the cortical neuron currents, it is found that indented edges can be detected after $20\mathrm{ ms}$ (time delay to convey contact information to the cortex). (**B**) The superior performance of the biomimetic decoder in recognizing edge stimuli indented at a different position on the simulated skin. (**C**) Confusion matrix for two classifiers, the biomimetic and the KNN classifiers. (**D**) Simulated receptive fields of SA-I and RA-I for 4 different scales (SD (mm)) based on the Gaussian distribution of the innervated mechanoreceptors. (**E**) Exploring the effect of afferent receptive field size on the temporal and spatial coding of the cortical neurons. Network Performance for spike counting (green) and spike timing (blue) when edge stimulus is scanned across the skin for $30 ms$ (left) and $50 ms$ (right) after contact. (F) Network Performance for spike counting (green) and spike timing (blue) for $30 ms$ (left) and $50 ms$ (right) after contact.

**Figure 9:**
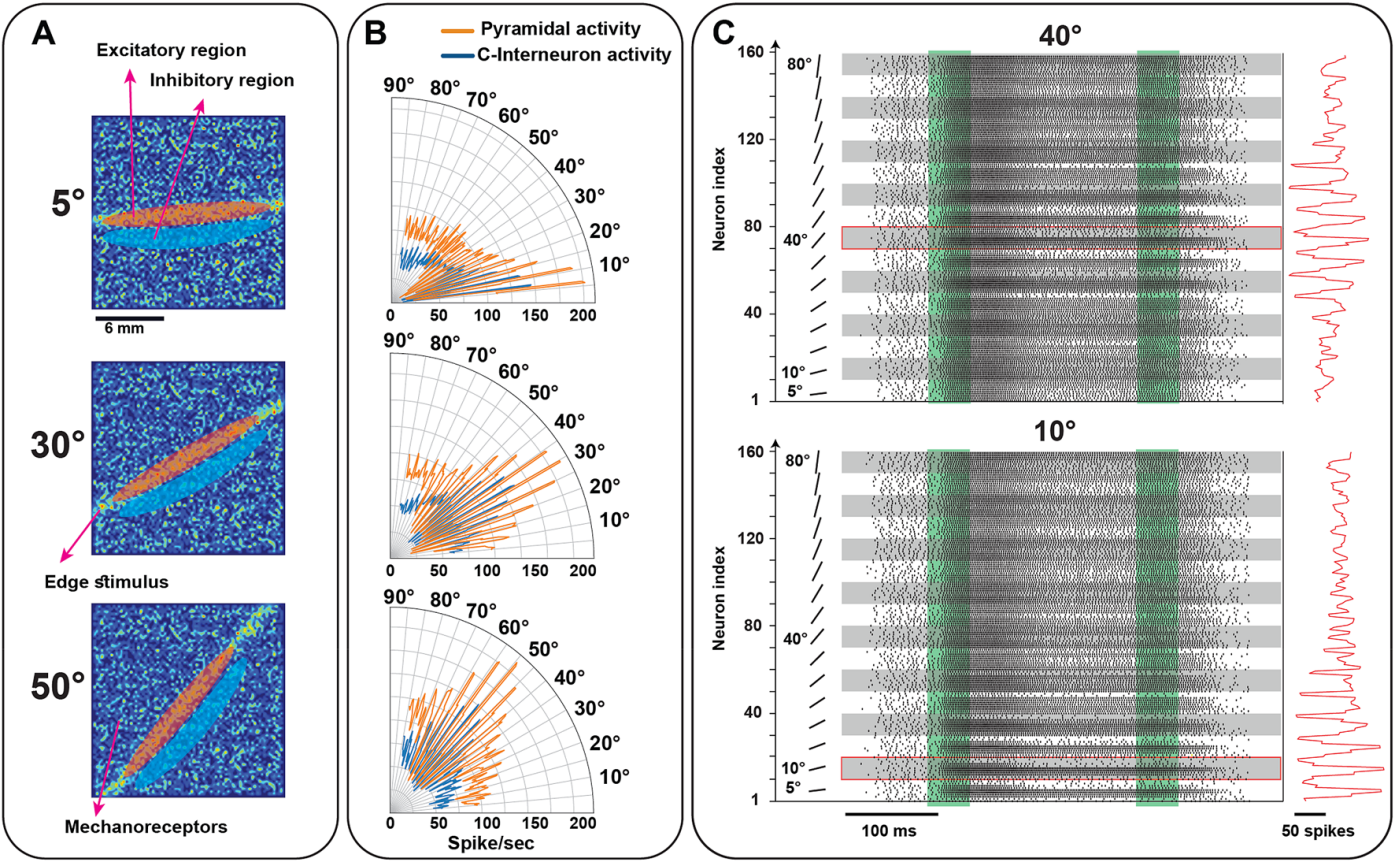
The cortical neuron receptive fields with excitatory and inhibitory sub-regions on the skin. (**A**, **B**) Receptive fields of cortical neurons are located at different positions across the skin and neurons selectively respond to the orientation stimulus. Activation of excitation areas on the skin increases the cortical firing, on the other hand, activation of inhibitory areas leads to a decrease in the spontaneous firing. (**C**). The firing response of 160 orientation-sensitive neurons in area 3b to 10° and 40° indented edge stimuli. Each group of the ten neurons is sensitive to one orientation which location of their receptive fields on the skin is different. The green areas show the onset and end phases of indentation.

In the last layer, the responses of cortical neurons to orientation stimuli are simulated. The obtained results are in agreement with the experimental results reported in the literature^35,37^ when an edge indents the skin. For simplicity, in our simulations, cortical receptive fields consist of two sub-regions on the skin (excitatory and inhibitory), and they are located close to each other and are tuned for a specific orientation. These receptive fields are distributed over the skin, therefore, edge orientation can be recognized accurately and rapidly by the cortical neurons even when the position of the edge stimulus on the skin is changing. Indeed, cortical neurons in area 3b are sensitive to specific orientations. Specifically, when an edge indents the skin, the input currents of orientation-tuned cortical neurons are raised based on the spatial coincidence between the receptive field sensitive zones of cortical neurons and the edge orientation on the skin. To better understand the function of the cortical neurons, the excitatory and inhibitory currents are plotted (Fig. 8A). It is evident that edge orientation can quickly be decoded using the winner-take-all algorithm. As shown in Fig. 8A, the current amplitudes of the orientation-tuned cortical neurons are increased after $20 ms$ to distinguish edge stimuli, which is compatible with the conduction delay to the cortex in neurophysiological observations^37^. To investigate how the biomimetic decoder recognizes edge orientations indented at different skin positions, we considered three locations with 1.2 mm spacing along the y-axis on the simulated patch of skin. For each trial, the stimulus was applied randomly to one of these locations. The orientation is decoded based on the “winner-take-all” approach (biomimetic decoder). Furthermore, to compare the performance with a common machine learning algorithm, the PCA for feature reduction and the KNN as the classifier are used. The spike count is used as the input features. To explore the evolution of decoding performance over time, the procedure was repeated for 60 progressively expanding time windows, ranging from $0$ to $180 ms$ after stimulus onset (Fig. 8B). As can be seen in Fig. 8B, the biomimetic decoder has better performance and achieves 97% accuracy around $180 ms$. Confusion matrices for both decoders are shown for the $180 ms$ time window (Fig. 8C). Another interesting simulation is to explore the effect of afferents’ receptive field size on the orientation recognition by the cortical neurons. The procedure for forming the receptive fields has been described in the Methods section. Here, we examine how the scanned and indented edge stimuli are encoded by the population of cortical spiking neurons when the standard deviation (SD) of the mechanoreceptor distribution is changed (Fig. 8D). Interestingly, it is observed that the larger receptive fields of afferents convey more information about the scanned edge in the first spikes of population-level of cortical neurons for both spike timing and spike count protocols (Fig. 8E). Conversely, in the indented edge experiment, the larger receptive fields make the orientation detection worse (Fig. 8F). The responses of all layers to an edge indentation stimulus are shown and summarized in Movie S1. Figure 9A illustrates sample receptive fields with excitatory and inhibitory sub-regions on the skin. An excitatory (inhibitory) sub-region indicates that any indentation at that location gives rise to an increase (decrease) in neuronal firing. This figure shows that the intensity of neural firing indicates the edge orientation. In this way, as the degree of spatial coincidence between the neuron sensitive zones and tissue deformations caused by edge indentation increases, the neural firing also increases. For a given neuron, some edge orientations show more spatial coincidence than others and therefore yield stronger responses (Fig. 9B and Movie S2). Raster plots of cortical neurons are depicted for two edge orientations (10° and 40°); the input stimulus is recognized by the highest spiking rate (Fig. 9C).

## Conclusion

The functional modeling of the tactile pathway from the cutaneous mechanoreceptors (first layer), to the cuneate nucleus (second layer) up to the somatosensory area 3b (third layer), provides a mechanistic tool for understanding the role of different neuronal networks in tactile information processing. The current research highlights the importance of each stage in neuronal population coding in the detection of edge orientation. It also provides a deeper understanding of how the response of cortical neurons to edge stimuli changes as the mechanoreceptor innervation mechanisms and receptive fields are changed. The simulated spiking neural networks are functionally compatible with physiological observations across a wide range of conditions sampled from literature. Indeed, many recent neurophysiological findings have been embedded in the proposed model and its performance—based on spiking responses of cortical neurons—has been demonstrated for decoding of edge orientations.

One of the key features of the human fingertip is its ability to recognize edge orientation. In this way, it was illustrated that the random innervation of the mechanoreceptors by the primary afferents allows the encoding of orientation information through the spatiotemporal spiking pattern. This structure organizes a peripheral neural mechanism for extraction and then transmission of geometric features of the touched objects. The proposed hierarchical spiking neural network successfully discriminated edge orientation stimuli irrespective of edge location. It was shown that using the first spikes of cortical neurons; the orientation of stimuli (scanned or indented edge) was recognizable. The effect of afferent receptive field size was compared in two different experiments (scanned and indented edge). Orientation detection of the scanned edge stimuli in the first spikes of cortical neurons was improved when the afferents’ receptive field size was increased. Nevertheless, for the indented edge experiment, the situation was reversed and increasing the size of the afferents receptive field resulted in the reduction of correct detection. The findings showed that the importance of receptive field size depends on the specific tasks and experiments. Recent studies have shown that the main connections in neuronal pathways are formed during the developmental process^38–40^. However, the exact cortical dynamics and function have not been studied yet. Here, we investigated edge orientation detection through the cortical neurons as a biomimetic classifier. We showed that the intensity of a neuron’s response would signal edge orientation because its firing rate would increase with the degree of spatial coincidence between the neuron’s highly sensitive zones (excitatory region of receptive field) and the local skin deformations formed by edge indentation. That is, for a given neuron, some edge orientations exhibit more spatial coincidence than others and thus stronger responses are produced.

Also, the role of the inhibitory current which forms the lateral inhibition within the cuneate nucleus was studied. Indeed, the simulation results suggest that when lateral inhibition has increased the process of spike filtering is amplified. This leads to the reduction in “noise” within the system and hence the third-order neurons are activated by a strong and consistent signal. This also increases the spatial resolution of the receptive fields and gives them a more distinct border which improves discrimination between two separate points of simultaneous stimulation. Although other forms of lateral inhibition are also observed, the “feedforward” type of lateral inhibition is likely the most significant^41^. Various aspects of tactile sensitivity have been related to different forms of neuronal inhibitory function. Impaired reactions to tactile stimuli in children with autism spectrum disorder (ASD) are frequently reported symptoms. Indeed, impairments in filtering of or adaptation to tactile inputs have been described in ASD^42^. Under the assumption that the inhibitory mechanism is altered in ASD^43,44^, it can be suggested that dysfunction in lateral inhibition of the second layer of tactile processing or malfunction in the formation of the inhibitory sub-regions of the cortical neurons may also have a role. Understanding the specific mechanisms underlying sensory symptoms in ASD is still under investigation which may allow for more specific therapeutic approaches in the future.

The main limitation of the proposed spiking model is the lack of neural recordings for all network layers. Nevertheless, the model is based on the significant literature and published data for model building and validation. The proposed spiking network for a tactile system can be employed in the design and implementation of sensory neuroprostheses applications^45–48^. Additionally, the broad significance of this work is that the biomimetic tactile sensing and edge encoding are useful in robotic applications for shape recognition and object grasping and palpation^49–51^.

## Methods

### Mechanoreceptor and afferent models

To simulate the mechanoreceptor grid, the physiological information from the literature was considered. The SA-I fibers constitute about 25% of all tactile fibers innervating the hand. They are divided into multiple branches near the skin surface to reach the Merkel discs, which are distributed over different fingerprint ridges. This branching and innervation create a receptive field covering an area of about 10 ${mm}^{2}$ on average for each SA-I afferent^8^. Afferent parameters are reported in Table 2. In response to a skin indentation, the activated Merkel discs within the receptive field send their partial information via the SA-I fibers which produce sustained spiking responses that slowly decrease over time (Fig. 1E). The RA-I afferents form around 40% of all tactile fibers innervating the hand. They are split into branches to relay signals from multiple Meissner corpuscles. Typically, the RA-I receptive field size is a bit larger than the SA-I receptive field. They respond to mechanoreceptor gird deformation^52,53^, therefore when the skin does not move, no spike is generated.

**Table 2:** Afferent properties used in the simulations^8^.

Afferents	Receptive field size (${{\varvec{m}}{\varvec{m}}}^{2}$)	Density/${{\varvec{c}}{\varvec{m}}}^{2}$	Number of afferents on the skin ($12\mathbf{m}\mathbf{m}\mathbf{*}12\mathbf{m}\mathbf{m}$)	${{\varvec{\sigma}}}_{{\varvec{d}}}$ (mm)
SA-I	$10$	$70$	$100$	0.3
RA	$12$	$140$	$196$	0.39

For modeling and simulation of the tactile afferents, we considered a grid of mechanoreceptors (80*80) as a patch of skin in which individual mechanoreceptors are located at 0.15 mm intervals^5^. A population of 296 first-order neurons were modeled according to biological observations and were consisted of 100 SA-I and 196 RA-I with overlapping innervation territories. Each primary afferent innervates 28 mechanoreceptors on average^11^ and is randomly weighted between 0.1 and 1. This branching leads to having complex receptive fields with multiple hotspots. To create the receptive field of SA-I afferents, we mapped a 10*10 network (100 SA-I neurons) on the 80*80 mechanoreceptor grid to cover all areas. The center of each section from the 10*10 mapped network is considered as a middle point of an individual receptive field. Then, 28 mechanoreceptors were chosen randomly using a Gaussian distribution in the $x$-axis and $y$-axis (${\sigma }_{d}$ in Table 2) and randomly weighted between 0.1 and 1. For RA-I afferents, we mapped a 14*14 network (196 RA-I neurons) on the 80*80 mechanoreceptor grid. The RA-I receptive field was generated similarly to the SA-I afferents. In total, 100 and 196 matrices with dimensions of 80 by 80 for SA-I and RA-I afferents were created, respectively.

The applied force distribution over the skin patch is modeled using a two-dimensional Gaussian function, and thus the output of the individual mechanoreceptor at location $\left(x.y\right)$ is shown as follows^54^:

1 $$\mathrm{f}\left(\mathrm{x},\mathrm{y}\right)=\mathrm{F}*\mathrm{exp}\left(-\left(\frac{{\left(\mathrm{x}-{\mathrm{x}}_{0}\right)}^{2}}{2*{\sigma }_{x}^{2}}+\frac{{\left(\mathrm{y}-{\mathrm{y}}_{0}\right)}^{2}}{2*{\sigma }_{y}^{2}}\right)\right)$$

where F is amplitude, (x_0_, y_0_) is center of pressure, and $f\left(x,y\right)$ is the perceived force for each mechanoreceptor at the location $\left(x,y\right)$. ${\sigma }_{x}= {\sigma }_{y}=0.05$ are standard deviation. The output of Eq. (1) is shown in Fig. S2A.

The Izhikevich model was used to reproduce the adaptation dynamics of the mechanoreceptors^55^. Indeed, this neuron model is easily able to generate different spiking patterns by simple parameter adjustment^56^. For the Izhikevich model, the membrane potential, *v,* and the adaptation variable, *u*, were updated via the following differential equations which were discretized using Euler’s method with the discretizing step = $0.1 ms$:

2 $$\frac{dv}{dt}=0.04{v}^{2}+5v+140-u+{k}_{SA}{I}_{SA}+{k}_{RA}{I}_{RA} $$

3 $$\frac{du}{dt}=a(bv-u)$$

4 $$if\, v\ge 30 mV.\, then \left\{\begin{array}{c} v \leftarrow c\\ u \leftarrow u+d\end{array}\right.$$

The values of the parameters a*, b, c,* and *d* are reported in Table 3. In Eq. (2), for the SA-I model ${k}_{SA}=1 \mathrm{and }{k}_{RA}=0$ and for the RA-I model ${k}_{SA}=0 \mathrm{and }{k}_{RA}=1$.

**Table 3:** Parameter values of the spiking model of SA-I and RA-I used in the simulations. Izhikevich neuron model parameters (a, b, c, d) of primary afferents are taken from^34^.

Parameter	Primary afferents	CN and cortical neurons	Parameter	Value
${\varvec{a}}$	$0.02\boldsymbol{ }{\mathbf{s}}^{-1}$	$0.1\boldsymbol{ }{\mathbf{s}}^{-1}$	${{\varvec{\tau}}}_{{\varvec{d}}\_{\varvec{S}}{\varvec{A}}}\boldsymbol{ }\mathbf{a}\mathbf{n}\mathbf{d}\boldsymbol{ }{{\varvec{\tau}}}_{{\varvec{R}}{\varvec{A}}}$	$30\boldsymbol{ }\mathbf{m}\mathbf{s}$
${\varvec{b}}$	$0.2$	$0.2$	${{\varvec{\tau}}}_{{\varvec{r}}\_{\varvec{S}}{\varvec{A}}}$	$5\boldsymbol{ }\mathbf{m}\mathbf{s}$
${\varvec{c}}$	$-65\boldsymbol{ }\mathbf{m}\mathbf{V}$	$-65\boldsymbol{ }\mathbf{m}\mathbf{V}$	${{\varvec{k}}}_{1}$	$0.05$
${\varvec{d}}$	$8\boldsymbol{ }\mathbf{m}\mathbf{V}$	$6\boldsymbol{ }\mathbf{m}\mathbf{V}$	${{\varvec{k}}}_{2}$	$3$
			${{\varvec{k}}}_{3}$	$2$

For the static SA-I model:

5 $${I}_{SA}={k}_{1}{I}_{in}$$

For the dynamic SA-I model:

6 $${\tau }_{d\_SA}\frac{d{I}_{SA}}{dt}=x-{I}_{SA}$$

7 $${\tau }_{r\_SA}\frac{dx}{dt}=({k}_{2}\frac{d{I}_{in}}{dt}+{k}_{1}{I}_{in}-x)$$

For the RA-I model:

8 $${\tau }_{RA}\frac{d{I}_{RA}}{dt}={k}_{3}|\frac{d{I}_{in}}{dt}|-{I}_{RA}$$

where ${\tau }_{r\_SA}$ and ${\tau }_{d\_SA}$ are the rise and decay time of the proposed SA-I model, respectively. $x$ is an auxiliary variable that is used in the proposed SA-I model.${I}_{in}$ is the input current to the network and, ${\tau }_{RA}$ is the time constant for the RA-I model.

Considering neurophysiological observation, the proposed spiking neuronal network of CN is comprised of two subpopulations of PNs and INs. Individual sub-populations are modeled by a random recurrent connection of spiking neurons with a connection probability of 0.2. Primary afferents (PA) make excitatory connections to both PN and IN spiking neuronal networks. Specifically, if a primary afferent excites a PN, neighboring afferents will excite INs to inhibit the activated PN (Fig. 5A). In this way, the concept of lateral inhibition is modeled. The simulated network model has 296 sensory input channels (PAs) that innervate each CN (PN and IN subpopulation). It should be pointed out that this number of afferents is less than the biological observation, which is around 1000s of PAs per CN^57^. To make the decoding problem more biologically relevant, in the simulations, we added intrinsic noise in the simulated pathways and considered the conduction velocities based on the synaptic delay for each layer.

Relying on prior neurophysiological findings, the local circuit in the somatosensory cortex area 3b was modeled by a neuronal network of 320 (160 PYs and 160 c-INs) spiking neurons. The cortical spiking network receives input from the PN subpopulation in the CN and is composed of a subpopulation of c-INs and a subpopulation of PYs. A different group of neurons in the cortical neuronal network are selective to different orientations. For each group, 10 PY and 10 c-IN was considered.

### Synaptic inputs

The CN and cortical neurons were also modeled using the Izhikevich model. The dynamic equation of excitatory (AMPA) and inhibitory (GABA) currents are as follow:

9 $${\tau }_{dA}\frac{d{I}_{AMPA}}{dt}=-{I}_{AMPA}+{x}_{A}$$

10 $${\tau }_{rA}\frac{d{x}_{A}}{dt}=-{x}_{A}+{W}_{k\_ex}\sum_{PAs}\delta \left(t-{t}_{ex}^{*}-{\tau }_{L}\right)$$

11 $${\tau }_{dG}\frac{d{I}_{GABA}}{dt}=-{I}_{GABA}+{x}_{G}$$

12 $${\tau }_{rG}\frac{d{x}_{G}}{dt}=-{x}_{G}+{W}_{k-in}\sum_{in}\delta (t-{t}_{in}^{*}-{\tau }_{L})$$

where ${I}_{AMPA}$ $and {I}_{GABA}$ are the excitatory and inhibitory synaptic currents received by the post-synaptic neuron, respectively. ${t}_{ex}^{*}$ and ${t}_{in}^{*}$ are the spike time received from pre-synaptic to the post-synaptic neuron. The latency of the post-synaptic currents is ${\tau }_{L}=1 ms$^58^. Synaptic constant times are ${\tau }_{dA}=0.4 ms$, ${\tau }_{rA}=2 ms$, ${\tau }_{dG}=0.25 ms$, ${\tau }_{rG}=5 ms$^58^. In our simulations, these values are multiplied by 7. ${W}_{k}$ is the synaptic weight of the connection between a pair of neurons.

### Cortical receptive fields

Cortical neurons have specific receptive fields that are feature selective. The cortical receptive fields consist of complex spatial excitatory and inhibitory sub-regions on the skin. To create a receptive field for individual PYs, the synaptic plasticity of the selected neuron is activated to strengthen excitatory and inhibitory connections related to the tuned orientation. After that, synaptic weights are fixed for all experiments. For each neuron in the cortical layer, the cumulative spike counts of all afferents are weighted based on their receptive fields and then are summed to determine the cortical neuron response^35^. We allocated a group of 10 neurons per orientation in the cortical neuronal network and their receptive fields were distributed across the skin. Since we simulated 16 different angles, 160 spiking neurons were used for modeling the cortical area. When the excitatory (inhibitory) sub-region is activated on the skin, PY connections become stronger (c-IN inhibits the PYs).

### Biomimetic decoder

The biomimetic decoder mimics the behavior of neuronal circuits in the somatosensory cortex^17^. The decoded orientation corresponds to the highest firing neuronal group. The biomimetic decoder gives a scalar value by counting the spikes of each cortical neurons within the neuronal group.

### Experiment simulation

Simulated edges are indented at the center of the patch of skin for $300 ms$, including a $50 ms$ on-ramp, a $200 ms$ hold phase, and a $50 ms$ off-ramp. Equation (13) simulates an edge to define specific orientation which is determined by parameter $\theta$. The simulated edge vertically indents the simulated patch of skin (80*80 mechanoreceptor grid):

13 $$Edge(\theta )=\mathrm{exp}(-{(x*\mathrm{sin}\left(\theta \right)+y*\mathrm{cos}(\theta )}^{2})/(2*{w}^{2}))$$

$x and y$ are coordination of mesh grid (80*80) and each axis is between − 1 and 1. $w=0.05$ is the edge width and finally, $\theta$ is the orientation of the simulated edge. The output of Eq. (13) is shown in Fig. S2B for $\theta =45^\circ$. To simulate the dynamics of a scanned edge, the speed of moving edge across the patch of skin is $24 mm/s$ (12 $mm$ in 500 $ms$).

### Noise

To improve the biological plausibility of the spiking neural model, a random fluctuation in the membrane potential is added (as intrinsic noise) to the neuron responses^3^. In this way, background activity is included in the population of neurons for each layer. The random noise is specified by a stochastic differential equation using a Gaussian random variable with mean 0 and standard deviation 1. Furthermore, to mimic the small variations in the presentation of stimulus and movement of skin in different trials, jitter in the depth (1 mm ± 0.5 mm SD) has been included. The numbers are used from^3^.

### Spike timing analysis at the population level

To address how much spike timing is informative to decode edge orientation, we used the spike train distance metric as defined by VPd which fully has been explained in^59^.

### PCA and classification

Principal component analysis^60^ is used to reduce the dimensionality of a feature space (using sklearn in Python). The feature space of simulated neural responses has high dimensionality and therefore is impossible to visualize. PCA gives the best possible representation of a p-dimensional dataset in $\mathrm{z}$ dimensions $(\mathrm{z}<\mathrm{p})$ by maximizing variance in $\mathrm{z}$ dimensions. In our simulations z = 3. Spiking responses are categorized across different time windows, starting from the first spike. For classification of the orientations, a KNN classifier with $k=5$ was used and decoding performance was evaluated using fivefold cross-validation. In this way, the process was repeated five times, and each of the five sets was used as the validation set once.

## Supplementary Information

Supplementary Information.

Supplementary Movie 1.

Supplementary Movie 2.

## References

[1] Talbot WH, Darian-Smith I, Kornhuber HH, Mountcastle VB (1968). The sense of flutter-vibration: comparison of the human capacity with response patterns of mechanoreceptive afferents from the monkey hand. J. Neurophysiol..

[2] Vallbo Å, Hagbarth K-E (1968). Activity from skin mechanoreceptors recorded percutaneously in awake human subjects. Exp. Neurol..

[3] Saal HP, Delhaye BP, Rayhaun BC, Bensmaia SJ (2017). Simulating tactile signals from the whole hand with millisecond precision. Proc. Natl. Acad. Sci..

[4] Pruszynski JA, Johansson RS (2014). Edge-orientation processing in first-order tactile neurons. Nat. Neurosci..

[5] Hay, E. & Pruszynski, J. A. Orientation processing by synaptic integration across first-order tactile neurons. *bioRxiv* 396705 (2018). 10.1371/journal.pcbi.1008303 PMC7710081 33264287

[6] Thakur PH, Fitzgerald PJ, Lane JW, Hsiao SS (2006). Receptive field properties of the macaque second somatosensory cortex: nonlinear mechanisms underlying the representation of orientation within a finger pad. J. Neurosci..

[7] Yau JM, Pasupathy A, Fitzgerald PJ, Hsiao SS, Connor CE (2009). Analogous intermediate shape coding in vision and touch. Proc. Natl. Acad. Sci..

[8] Delhaye BP, Long KH, Bensmaia SJ (2018). Neural basis of touch and proprioception in primate cortex. Compr. Physiol..

[9] Johansson RS, Vallbo A (1979). Tactile sensibility in the human hand: relative and absolute densities of four types of mechanoreceptive units in glabrous skin. J. Physiol..

[10] Corniani G, Saal HP (2020). Tactile innervation densities across the whole body. J. Neurophysiol..

[11] Nolano M (2003). Quantification of myelinated endings and mechanoreceptors in human digital skin. Ann. Neurol..

[12] Rongala UB (2018). Intracellular dynamics in cuneate nucleus neurons support self-stabilizing learning of generalizable tactile representations. Front. Cell. Neurosci..

[13] Jörntell H (2014). Segregation of tactile input features in neurons of the cuneate nucleus. Neuron.

[14] Jones EG (2000). Cortical and subcortical contributions to activity-dependent plasticity in primate somatosensory cortex. Annu. Rev. Neurosci..

[15] Abraira VE, Ginty DD (2013). The sensory neurons of touch. Neuron.

[16] Reed JL (2008). Widespread spatial integration in primary somatosensory cortex. Proc. Natl. Acad. Sci..

[17] DiCarlo JJ, Johnson KO, Hsiao SS (1998). Structure of receptive fields in area 3b of primary somatosensory cortex in the alert monkey. J. Neurosci..

[18] Bensmaia SJ, Denchev PV, Dammann JF, Craig JC, Hsiao SS (2008). The representation of stimulus orientation in the early stages of somatosensory processing. J. Neurosci..

[19] Pei Y-C, Hsiao SS, Craig JC, Bensmaia SJ (2010). Shape invariant coding of motion direction in somatosensory cortex. PLoS Biol..

[20] Chung S, Li X, Nelson SB (2002). Short-term depression at thalamocortical synapses contributes to rapid adaptation of cortical sensory responses in vivo. Neuron.

[21] Katz Y, Heiss JE, Lampl I (2006). Cross-whisker adaptation of neurons in the rat barrel cortex. J. Neurosci..

[22] Reed JL, Qi H-X, Kaas JH (2011). Spatiotemporal properties of neuron response suppression in owl monkey primary somatosensory cortex when stimuli are presented to both hands. J. Neurosci..

[23] Brouwer GJ, Arnedo V, Offen S, Heeger DJ, Grant AC (2015). Normalization in human somatosensory cortex. J. Neurophysiol..

[24] Saal HP, Harvey MA, Bensmaia SJ (2015). Rate and timing of cortical responses driven by separate sensory channels. Elife.

[25] Sripati AP, Yoshioka T, Denchev P, Hsiao SS, Johnson KO (2006). Spatiotemporal receptive fields of peripheral afferents and cortical area 3b and 1 neurons in the primate somatosensory system. J. Neurosci..

[26] Goodman DF, Brette R (2009). The brian simulator. Front. Neurosci..

[27] Gerling GJ, Wan L, Hoffman BU, Wang Y, Lumpkin EA (2018). Computation predicts rapidly adapting mechanotransduction currents cannot account for tactile encoding in Merkel cell-neurite complexes. PLoS Comput. Biol..

[28] Lesniak DR (2014). Computation identifies structural features that govern neuronal firing properties in slowly adapting touch receptors. eLife.

[29] Woo S-H, Lumpkin EA, Patapoutian A (2015). Merkel cells and neurons keep in touch. Trends Cell Biol..

[30] Maksimovic S (2014). Epidermal Merkel cells are mechanosensory cells that tune mammalian touch receptors. Nature.

[31] Woo S-H (2014). Piezo2 is required for Merkel-cell mechanotransduction. Nature.

[32] Valero, M. R., Hale, N., Tang, J. & Jiang, L. A comprehensive mechanotransduction model for tactile feedback based on multi-axial stresses at the fingertip-contact interface. In *2017 IEEE World Haptics Conference (WHC)* 43–47 (2017).

[33] Salimi-Nezhad N, Amiri M, Falotico E, Laschi C (2018). A digital hardware realization for spiking model of cutaneous mechanoreceptor. Front. Neurosci..

[34] Oddo CM (2017). Artificial spatiotemporal touch inputs reveal complementary decoding in neocortical neurons. Sci. Rep..

[35] Delhaye BP, Xia X, Bensmaia SJ (2019). Rapid geometric feature signaling in the simulated spiking activity of a complete population of tactile nerve fibers. J. Neurophysiol..

[36] Victor JD, Purpura KP (1996). Nature and precision of temporal coding in visual cortex: a metric-space analysis. J. Neurophysiol..

[37] Callier T, Suresh AK, Bensmaia SJ (2019). Neural coding of contact events in somatosensory cortex. Cereb. Cortex.

[38] Rao S, Hansel D, van Vreeswijk C (2019). Dynamics and orientation selectivity in a cortical model of rodent V1 with excess bidirectional connections. Sci. Rep..

[39] Shatz CJ (1990). Impulse activity and the patterning of connections during CNS development. Neuron.

[40] Beul SF, Goulas A, Hilgetag CC (2018). Comprehensive computational modelling of the development of mammalian cortical connectivity underlying an architectonic type principle. PLoS Comput. Biol..

[41] Li L-Y (2014). A feedforward inhibitory circuit mediates lateral refinement of sensory representation in upper layer 2/3 of mouse primary auditory cortex. J. Neurosci..

[42] Puts NA, Wodka EL, Tommerdahl M, Mostofsky SH, Edden RA (2014). Impaired tactile processing in children with autism spectrum disorder. J. Neurophysiol..

[43] Casanova MF (2004). White matter volume increase and minicolumns in autism. Ann. Neurol..

[44] DeLorey TM (2005). GABRB3 gene deficient mice: a potential model of autism spectrum disorder. Int. Rev. Neurobiol..

[45] D’Alonzo M, Engels L, Controzzi M, Cipriani C (2017). Electro-cutaneous stimulation on the palm elicits referred sensations on intact but not on amputated digits. J. Neural Eng..

[46] Okorokova EV, He Q, Bensmaia SJ (2018). Biomimetic encoding model for restoring touch in bionic hands through a nerve interface. J. Neural Eng..

[47] Osborn, L. *et al.* Targeted transcutaneous electrical nerve stimulation for phantom limb sensory feedback. In *2017 IEEE Biomedical Circuits and Systems Conference (BioCAS)* 1–4 (2017). 10.1109/biocas.2017.8325200 PMC8068407 33899051

[48] George J (2019). Biomimetic sensory feedback through peripheral nerve stimulation improves dexterous use of a bionic hand. Sci. Robot..

[49] Kappassov Z, Corrales J-A, Perdereau V (2015). Tactile sensing in dexterous robot hands. Robot. Auton. Syst..

[50] Gupta, A., Eppner, C., Levine, S. & Abbeel, P. Learning dexterous manipulation for a soft robotic hand from human demonstrations. In *2016 IEEE/RSJ International Conference on Intelligent Robots and Systems (IROS)* 3786–3793 (2016).

[51] Osborn, L., Nguyen, H., Kaliki, R. & Thakor, N. Live demonstration: prosthesis grip force modulation using neuromorphic tactile sensing. In *2017 IEEE International Symposium on Circuits and Systems (ISCAS)* 1–1 (2017).

[52] Muniak MA, Ray S, Hsiao SS, Dammann JF, Bensmaia SJ (2007). The neural coding of stimulus intensity: Linking the population response of mechanoreceptive afferents with psychophysical behavior. J. Neurosci..

[53] Johansson RS, Landstro U, Lundstro R (1982). Responses of mechanoreceptive afferent units in the glabrous skin of the human hand to sinusoidal skin displacements. Brain Res..

[54] Delgado A (2017). Tactile control based on Gaussian images and its application in bi-manual manipulation of deformable objects. Robot. Auton. Syst..

[55] Oddo CM (2011). Roughness encoding in human and biomimetic artificial touch: spatiotemporal frequency modulation and structural anisotropy of fingerprints. Sensors.

[56] Izhikevich EM (2003). Simple model of spiking neurons. IEEE Trans. Neural Netw..

[57] Bengtsson F, Brasselet R, Johansson RS, Arleo A, Jörntell H (2013). Integration of sensory quanta in cuneate nucleus neurons in vivo. PLoS ONE.

[58] Mazzoni A, Panzeri S, Logothetis NK, Brunel N (2008). Encoding of naturalistic stimuli by local field potential spectra in networks of excitatory and inhibitory neurons. PLoS Comput. Biol..

[59] Vargas-Irwin CE, Brandman DM, Zimmermann JB, Donoghue JP, Black MJ (2015). Spike train SIMilarity Space (SSIMS): a framework for single neuron and ensemble data analysis. Neural Comput..

[60] Minka, T. P. Automatic choice of dimensionality for PCA. In *Advances in Neural Information Processing Systems* 598–604 (2001).

