# Supplementary material for: A functional spiking neuronal network for tactile sensing pathway to process edge orientation
Source: Sci Rep. 2021 Jan 14;11:1320. doi: 10.1038/s41598-020-80132-4 (PMC7809061; doi:10.1038/s41598-020-80132-4)
Supplement: Supplementary file 1 — Supplementary Information. [file 41598_2020_80132_MOESM1_ESM.docx]

**A Functional Spiking Neuronal Network for Tactile Sensing Pathway to Process Edge Orientation**

Adel Parvizi-Fard^1^, Mahmood Amiri^2*^, Deepesh Kumar ^3^, Mark M. Iskarous^4^, Nitish V. Thakor^3,4,5*^

^1^ Medical Biology Research Center, Kermanshah University of Medical Sciences, Kermanshah,
 Iran
^2^ Medical Technology Research Center, Kermanshah University of Medical Sciences, Kermanshah, Iran

^3^ SINAPSE Laboratory, National University of Singapore, Singapore

^4^ Department of Biomedical Engineering, Johns Hopkins University, Baltimore, MD, USA

^5^ Department of Biomedical Engineering, National University of Singapore, Singapore

*Corresponding author

Email:

[ma_amiri_bme@yahoo.com](mailto:ma_amiri_bme@yahoo.com) (M.A.)

[nitish@jhu.edu](mailto:nitish@jhu.edu) or [eletnv@nus.edu.sg](mailto:eletnv@nus.edu.sg) (N.V.T.)

**SUPPLEMENTARY FIGURES**

**
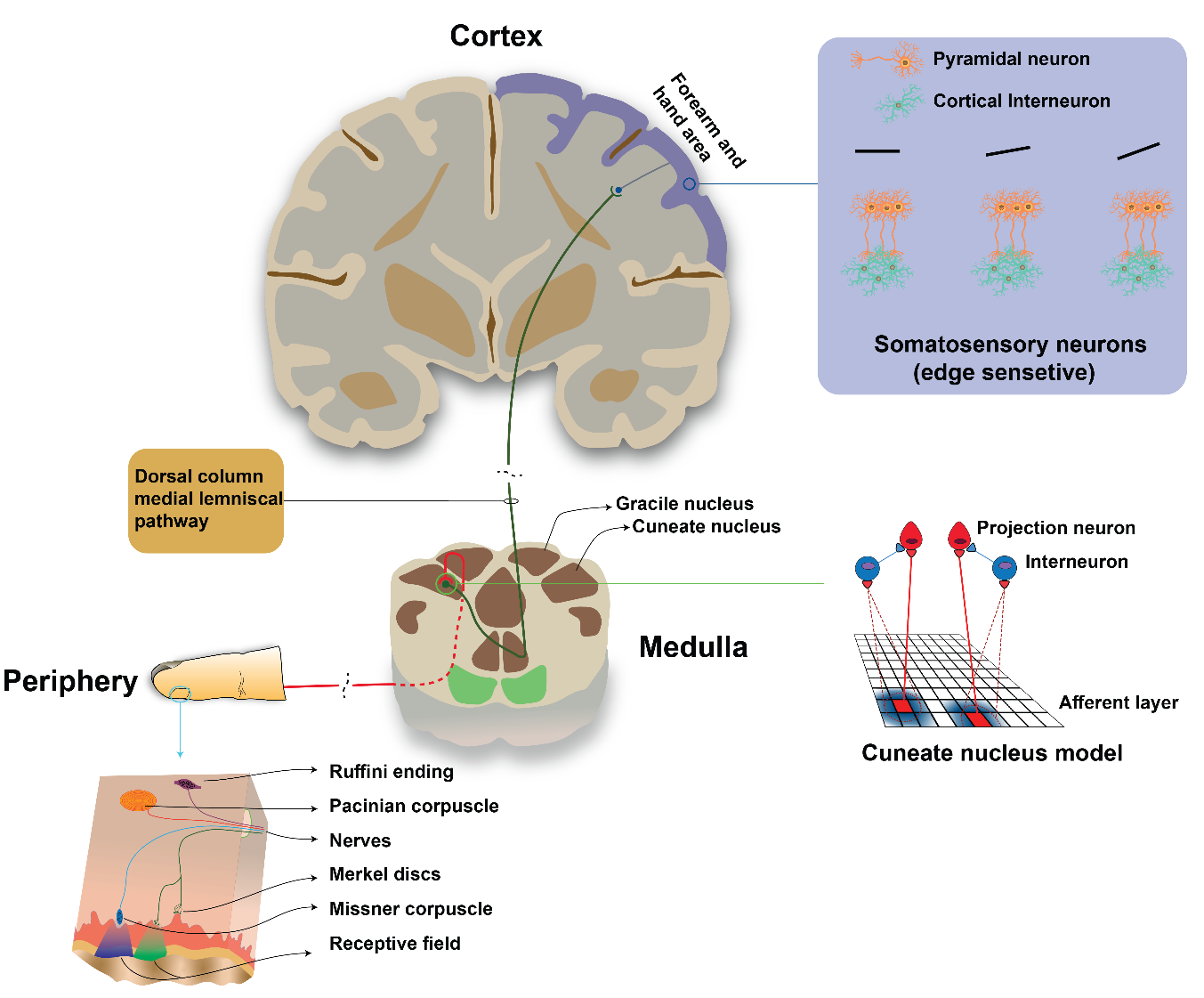
**

**Figure S1**. **The tactile processing pathway**. Three important stages of tactile information processing are shown. The first step is the primary afferents which randomly innervate the cutaneous mechanoreceptors. The second step takes place in the cuneate nucleus where lateral inhibition is applied for filtering of excessive firing. The third step includes the cortical neurons of the somatosensory area 3b which are orientation-sensitive.

**
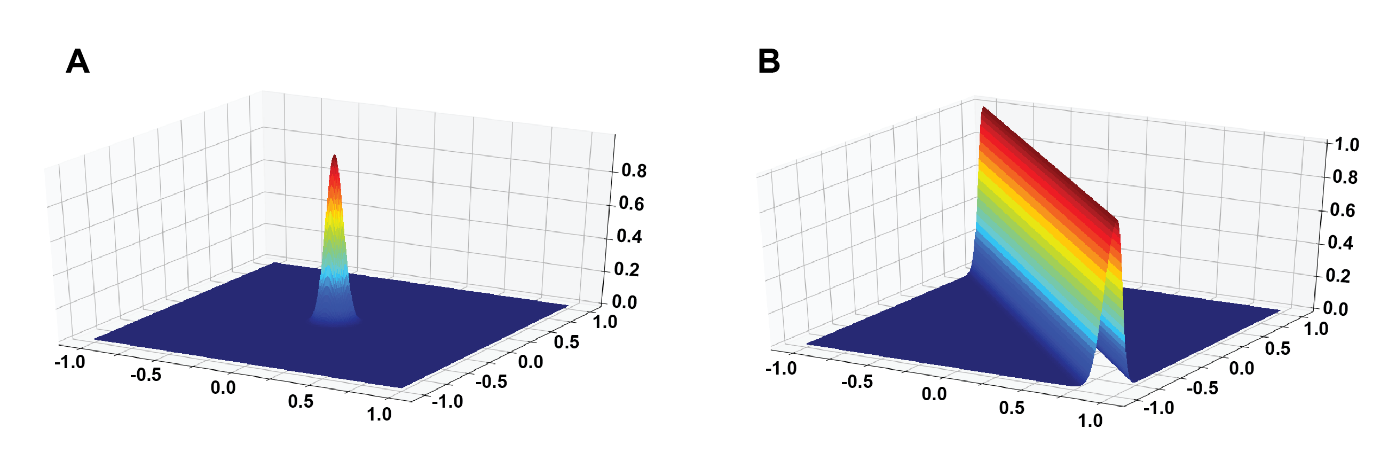
**

**Figure S2**. **Gaussian function and simulated edge stimulus**. (A) The Gaussian profile obtained from Eq (1) when $\sigma_{x}= \sigma_{y}=0.05$ . (B) The output of Eq (13) simulates the edge orientation stimulus (45°).

**
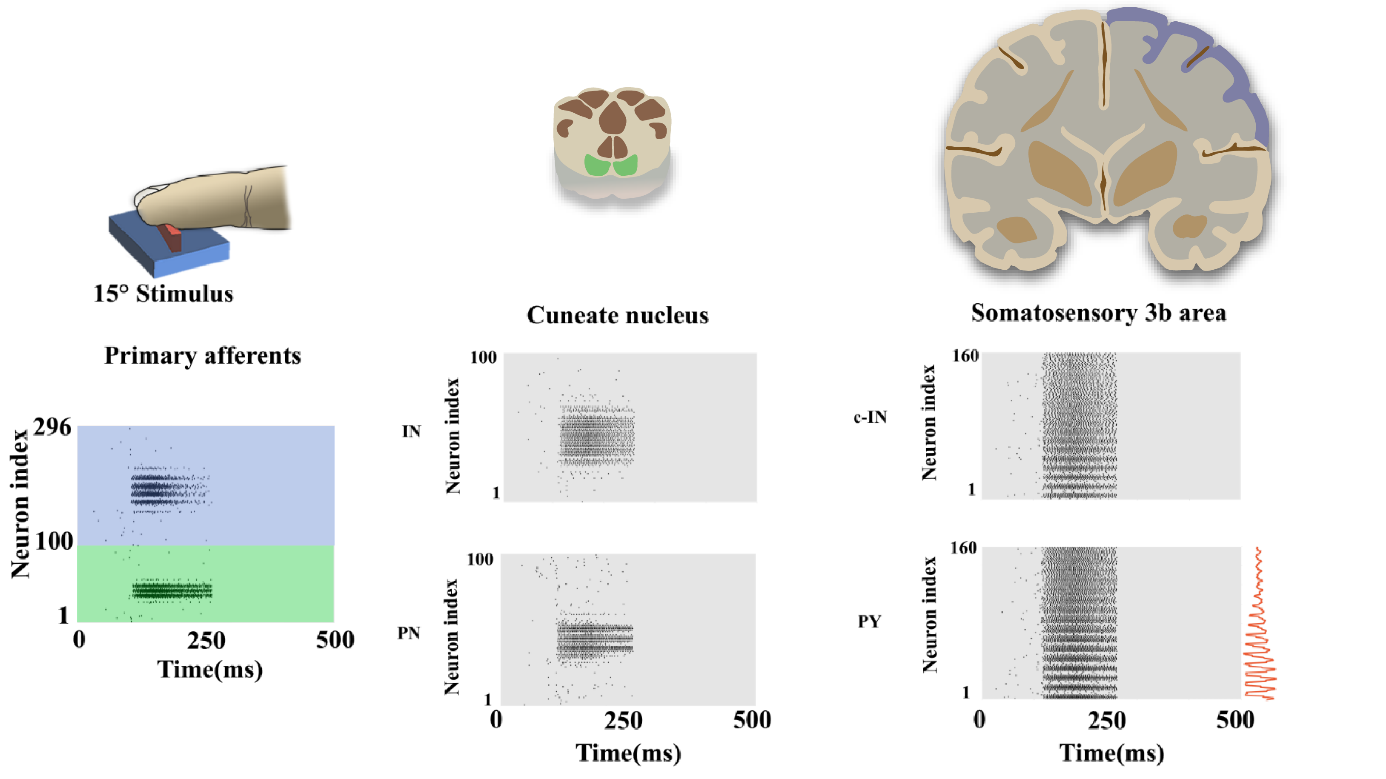
**

**Movie S1. Responses of three different layers to the 15° indented edge stimulus**. SA-I and RA-I firing are shown by green and blue colors. Spiking neuronal networks in the somatosensory cortex respond to the input stimulus so that the neurons with higher activities signal the correct orientation.


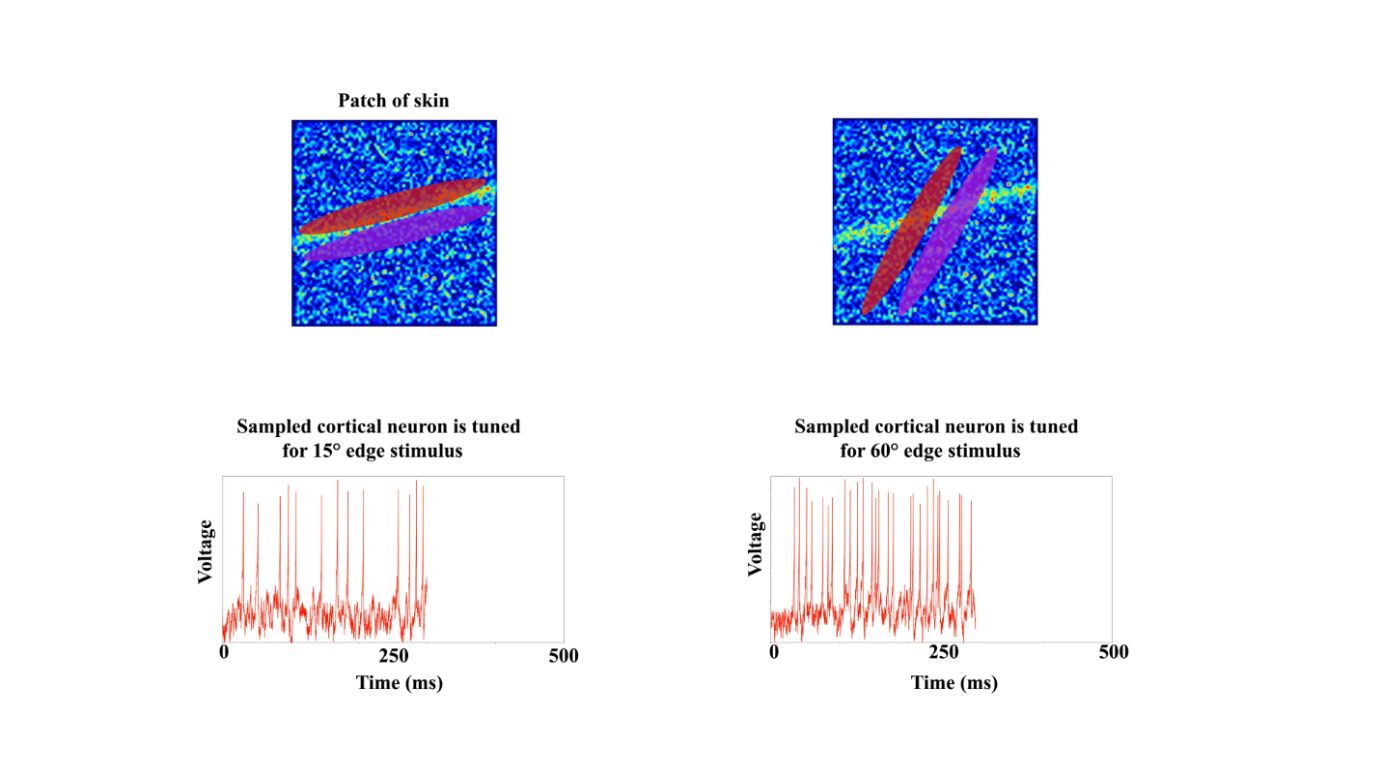


**Movie S2.** **Sampled cortical neurons respond to the scanned edge stimulus.** As the degree of spatial coincidence between the neuron’s receptive field and edge stimulus increases, higher neuronal firing is recorded.
